# Hybrid Open and Robot‐Assisted Excision of a Giant Multilocular Prostatic Cystadenoma With Unusual Dorsal Growth: A Case Report

**DOI:** 10.1155/criu/5630511

**Published:** 2026-10-28

**Authors:** Toshiki Kenuka, Yuki Kyoda, Manabu Okada, Fumiyasu Takei, Tetsuya Shindo, Kohei Hashimoto, Ko Kobayashi, Toshiaki Tanaka, Reiko Watanabe, Naoya Masumori

**Affiliations:** ^1^ Department of Urology School of Medicine Sapporo Medical University Sapporo Japan sapmed.ac.jp; ^2^ Department of Urology Hokkaido Social Work Association Obihiro Hospital Obihiro Japan dlut.edu.cn; ^3^ Tokachi Urological Clinic Otofuke Japan; ^4^ Department of Surgical Pathology School of Medicine Sapporo Medical University Sapporo Japan sapmed.ac.jp

**Keywords:** dorsal growth, giant multilocular prostatic cystadenoma, hybrid surgery, robot-assisted surgery

## Abstract

Large prostatic tumors are rare conditions that may present with atypical morphology on imaging studies and can pose substantial challenges in preoperative diagnosis. We encountered a case of giant multilocular prostatic cystadenoma (GMPC) with dorsal growth in which malignancy could not be excluded despite multiple biopsies demonstrating stromal hyperplasia. A 71‐year‐old man presented with lower abdominal pain associated with defecation, and imaging revealed a large pelvic mass with increased uptake on positron emission tomography. Because malignancy could not be excluded, radical prostatectomy was performed. The procedure was initiated robotically but required conversion to an open approach due to intraoperative difficulty. Histopathological examination of the 500‐g resected mass revealed stromal hyperplasia with multilocular cystic architecture, consistent with GMPC. Postoperatively, urinary function remained satisfactory, and no recurrence has been observed to date. This case highlights the diagnostic challenges associated with GMPC presenting with atypical growth patterns and demonstrates that complete excision is feasible with an appropriate surgical approach.

## 1. Introduction

Giant multilocular prostatic cystadenoma (GMPC) is a rare benign prostatic lesion that often poses diagnostic challenges preoperatively. We report a case of GMPC in which malignancy could not be excluded preoperatively and a hybrid robot‐assisted and open surgical approach was performed.

## 2. Case Presentation

A 71‐year‐old male presented with lower abdominal pain associated with defecation. His past medical history was notable for a left ureteral stone and a nonfunctioning left adrenal adenoma. He had undergone a transrectal prostate biopsy and transurethral resection of the prostate (TURP) 5 years earlier, both of which revealed no malignant findings. The prostate volume prior to TURP was 76.6 mL. His family history was unremarkable.

The patient presented to a local physician with lower abdominal pain during defecation. CT revealed a pelvic mass measuring 10 cm in diameter. MRI demonstrated a tumor measuring 11.4 × 10.4 × 9.4 cm (estimated volume approximately 580 mL) (Figure 1). Radiological diagnosis suggested stromal tumor of uncertain malignant potential (STUMP), a transitional zone tumor, or prostatic sarcoma. PET/CT showed FDG uptake within the tumor (SUV max 3.9), and malignancy could not be excluded. Laboratory tests revealed an elevated PSA level of 54.1 ng/mL. Two transrectal biopsies and a transurethral prostate biopsy performed at the previous hospital revealed stromal hyperplasia without malignancy. Re‐evaluation of the specimens at our institution confirmed stromal hyperplasia. Although pathology did not confirm malignancy, the imaging findings remained suspicious. MRI showed an indistinct boundary between the tumor and the prostate, suggesting that the lesion most likely originated from the prostate. Therefore, we determined that preserving the prostate and removing only the tumor could compromise oncological curability if the lesion proved to be malignant. For this reason, we judged that, at minimum, the prostate should be included in the tumor resection.

**Figure 1:**
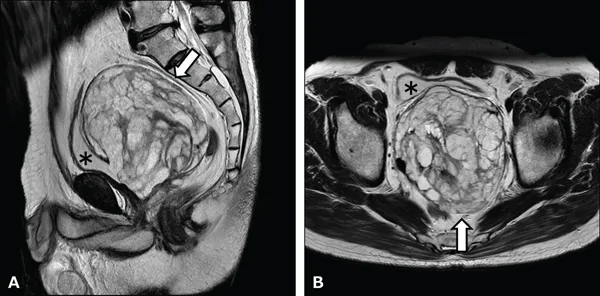
Plain magnetic resonance imaging of the abdomen and pelvis. (A) Sagittal and (B) axial views showing a large multilocular mass arising from the prostate gland. The arrows indicate the unusual dorsal growth of the tumor. The tumor demonstrates marked dorsal expansion (approximately 11 cm in maximum diameter), displacing the bladder (asterisk) anteriorly and compressing the rectum posteriorly.

Because intraoperative identification of the ureter was anticipated to be challenging, infrared illumination ureteral catheters (Infrared Illumination System; IRIS, Stryker) were placed in both ureters prior to the initiation of surgery. Robot‐assisted radical prostatectomy was commenced using the da Vinci Xi platform (Intuitive Surgical, Sunnyvale, California, United States). However, because of the substantial tumor volume, adequate visualization of the caudal and dorsal operative fields could not be achieved, making a robotic approach to the dorsal aspect technically unfeasible. Given that the tumor occupied the limited pelvic space, we anticipated that conversion to an open procedure would allow manual traction and provide a sufficiently wide operative field. Therefore, we proceeded with open conversion. Following specimen removal, the robotic system was redocked to complete the vesicourethral anastomosis using a running 3‐0 V‐Loc suture.

The total operative duration was 7 h and 1 min. Estimated blood loss was 1500 mL, and intraoperative transfusion consisted of two units of packed red blood cells and six units of fresh frozen plasma. The resected specimen weighed 500 g (Figure 2). The histological examination revealed no urethral architecture, suggesting this tumor is hemispheric. Microscopically nodular hyperplastic lesions of benign epithelial and stromal cells were observed with dilated bland cystic structure (Figure 3). Because the tumor was extremely large, representative sampling was performed from the solid, cystic, and stromal components. All sampled specimens demonstrated benign epithelial–stromal proliferation without atypia, mitotic figures, necrosis, or any features suggestive of sarcoma or carcinoma, supporting the interpretation that this was a benign tumor predominantly arising from the left lobe. Although complete microscopic examination of the entire 500‐g mass was not feasible, no malignant component was identified in the extensively sampled sections.

**Figure 2:**
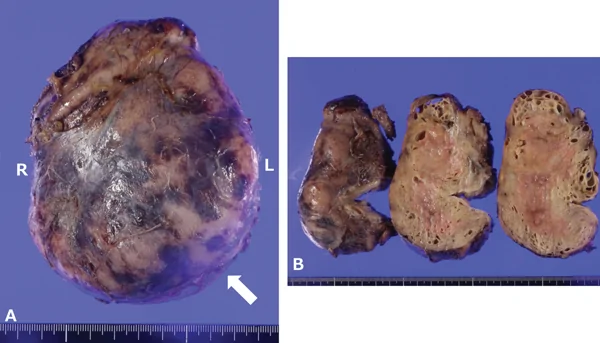
Gross photographs. (A) The dorsal surface of the resected specimen shows marked enlargement of the left lobe (arrow). (B) On the ventral aspect of the cut surface, the solid area showed nodular growth with clusters of small cysts.

**Figure 3:**
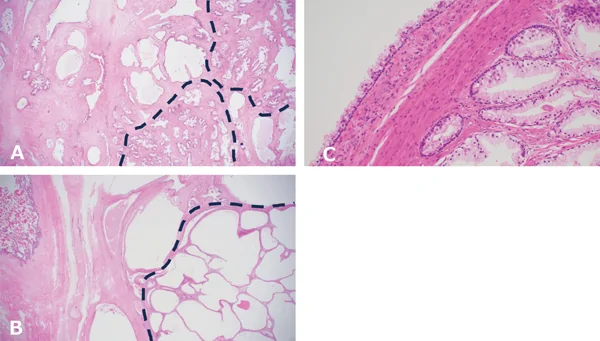
Histopathological findings. (A) Low‐power view showing a nodular proliferation of duct–acinar structures outlined by a dashed line (H&E, ×20). (B) Multilocular cystic architecture with variably dilated glandular and cystic spaces, outlined by a dashed line (H&E, ×40). (C) High‐power view showing crowded glandular structures lined by bland prostatic‐type epithelium without cytologic atypia (H&E, ×200).

On postoperative Day 7, cystography demonstrated leakage at the vesicourethral anastomosis, and the urethral catheter was therefore left in place. The leakage resolved by postoperative Day 65, at which point the catheter was removed. Serum PSA decreased to 0.032 ng/mL at 2 months postoperatively. At 3 months, the patient reported mild stress urinary incontinence (approximately two pads per day), which resolved completely by 8 months postoperatively. The lower abdominal pain present preoperatively disappeared immediately after surgery. Uroflowmetry at 3 months postoperatively showed a voided volume of 139.9 mL, a maximum flow rate of 19.9 mL/s, and a postvoid residual volume of 31 mL.

## 3. Discussion

Differential diagnoses of giant pelvic tumors include leiomyoma, leiomyosarcoma, liposarcoma, solitary fibrous tumor, extragastrointestinal stromal tumor, and neurofibroma [1]. Prostatic lesions include benign prostatic hyperplasia and STUMP. Definitions of giant prostatic adenoma vary: Some reports use > 200 mL, others > 500 mL [2, 3]. Our case (500 mL) meets these criteria. Giant pelvic tumors are often difficult to diagnose preoperatively [4], as in this case. Imaging contributed to the diagnostic challenge: Typical prostatic adenomas enlarge symmetrically around the urethra, whereas the tumor in this case grew dorsally, distorting the usual prostatic morphology (Figure 1). The radiologist interpreted the pelvic mass as arising from the peripheral region of the prostate or from an extraprostatic origin, with a malignant neoplasm considered the most likely diagnosis. Ultimately, pathology confirmed GMPC, but few previous reports describe such a large dorsal‐growing adenoma [5]. GMPC is an exceedingly rare benign prostatic lesion, with only a limited number of cases reported in the literature. This entity was first described by Maluf et al. [6] in 1991, characterized by a large multiloculated cystic tumor composed of glands and cysts lined by prostatic‐type epithelium, typically arising within the retrovesical or retroperitoneal region. In this case as well, microscopic examination (Figure 3) revealed variably dilated glandular and cystic structures lined by mixed prostatic‐type epithelium. These glands and cysts were further lined by cuboidal to columnar epithelial cells with a distinct and intact basal cell layer. Based on these findings, a diagnosis of GMPC was considered highly likely. Although the histological findings in our case were consistent with GMPC, the lesion also exhibited features that overlap with so‐called cystic epithelial–stromal tumors. In the current WHO Blue Book classification [7], cystic epithelial–stromal tumors are considered one of the related terms of STUMP; therefore, we may say that cystic epithelial–stromal tumors can be considered part of the STUMP spectrum, even when overt cytologic atypia or increased mitotic activity is absent. Although our case showed some morphological features overlapping with cystic epithelial–stromal tumors, which are classified as STUMP‐related entities in the WHO classification, it lacked histological features suggestive of STUMP, including stromal hypercellularity, cytologic atypia, mitotic activity, and necrosis. Therefore, the justification for placing this lesion within the STUMP spectrum remains limited, and GMPC is considered the most appropriate diagnosis. Nevertheless, the existence of recurrent GMPC reported in the literature [8] suggests that at least a subset of GMPCs may share a biological continuum with STUMP rather than representing a completely distinct entity.

The etiology of GMPC remains unclear. Chronic inflammation is believed to contribute to the pathogenesis of large prostatic tumors in general [9]. In this case, TURP and biopsy performed 5 years earlier may have triggered regrowth. Werneburg et al. reported rapid regrowth after repeated transurethral procedures, suggesting surgical trauma‐induced inflammation [10].

GMPC, a rare benign tumor, has been described under various pathological classifications, typically presenting with LUTS [11], but may represent a similar entity. Interestingly, the patient lacked LUTS, likely because prior TURP relieved bladder outlet obstruction and the dorsal growth spared the prostatic urethra (Figure 1).

Treatment options for GMPC include GnRH analogs [12], TURP, open simple prostatectomy, laparoscopic surgery, and robot‐assisted surgery [3]. HoLEP is often preferred for large adenomas [13, 14], with reports of safe enucleation after preoperative prostatic artery embolization in glands up to 696 mL [14]. However, in our case, dorsal growth limited endoscopic visualization, making transurethral or laparoscopic approaches difficult. PET uptake and MRI findings necessitated tumor resection. We elected to begin the procedure with a robot‐assisted approach because of its minimally invasive nature and its potential to preserve continence [3]. To the best of our knowledge, the largest tumor that has been safely resected via robot‐assisted surgery weighed 560 g; in that case, preoperative biopsy confirmed a diagnosis of prostate cancer [11]. Although previous reports have described the complete excision of GMPC using robot‐assisted surgery alone [15], those cases differed from ours in that they employed an extraperitoneal approach and involved slightly smaller adenoma volumes. In the present case, the large tumor volume and adhesions following treatment for prostatitis created technical difficulties that made completion by robot‐assisted surgery alone impractical. Nevertheless, despite the technical challenges, prolonged operative time, and the need for transfusion, the tumor was safely removed without resection of other organs, and the postoperative course was favorable.

Future efforts should focus on improving preoperative diagnostic accuracy through advanced imaging modalities and more extensive biopsy sampling, as well as reducing concerns about postoperative anastomotic leakage and shortening the duration of urethral catheterization.

## 4. Conclusion

We experienced a case of GMPC in which preoperative diagnosis was challenging. The tumor was completely removed by excising the prostate using a combination of robot‐assisted and open surgery. This approach may be feasible in highly selected patients, but considering the intraoperative and postoperative course in this case, its applicability and safety cannot be generalized.

**Nomenclature**
CT: computed tomography
FDG: fluorodeoxyglucose
GMPC: giant multilocular prostatic cystadenoma
GnRH: gonadotropin‐releasing hormone
HoLEP: holmium laser enucleation of the prostate
LUTS: lower urinary tract symptoms
MRI: magnetic resonance imaging
PET: positron emission tomography
PSA: prostate‐specific antigen
STUMP: stromal tumor of uncertain malignant potential
SUV: standardized uptake value
TURP: transurethral resection of the prostate

## Author Contributions

Conceptualization: Y.K. Methodology: Y.K. Investigation: T.K. and Y.K. Data curation: T.K. Writing—original draft: T.K. Writing—review and editing: Y.K. Supervision: M.O., F.T., T.S., K.H., K.K., T.T., R.W., and N.M.

## Funding

No funding was received for this manuscript.

## Disclosure

All authors have read and approved the final version of the manuscript. Dr. Yuki Kyoda had full access to all of the data in this study and takes complete responsibility for the integrity of the data and the accuracy of the data analysis.

## Consent

Written informed consent was obtained from the patient for publication of this case report and accompanying images.

## Conflicts of Interest

The authors declare no conflicts of interest.

## Data Availability

The data that support the findings of this study are available from the corresponding author upon reasonable request.

## References

[1] Takeda M. , Ide Y. , Tokuoka Y. , and Sasaki Y. , A Solitary Fibrous Tumor in the Pelvic Cavity, Journal of Japan Surgical Association. (2014) 75, no. 7, 2037–2042, 10.3919/jjsa.75.2037.

[2] Kawamura S. , Takata K. , Yoshida I. , and Matsui S. , A Case of Giant Prostatic hypertrophy, Hinyokika Kiyo. (1984) 30, no. 12, 1861–1866, 6085220. 6085220

[3] Fishman J. and Merrill D. , A Case of Giant Prostatic Hyperplasia, Urology. (1993) 42, no. 3, 336–337, 10.1016/0090-4295(93)90628-n. 7691015

[4] Herawi M. and Epstein J. , Specialized Stromal Tumors of the Prostate: A Clinicopathologic Study of 50 Cases, American Journal of Surgical Pathology. (2006) 30, no. 6, 694–704, 10.1097/00000478-200606000-00004, 16723846. 16723846

[5] Fan L. , Chang Y. , Shao I. , Wu K. F. , and Pang S. T. , Robotic Surgery in Giant Multilocular Cystadenoma of the Prostate: A Rare Case Report, World Journal of Clinical Cases. (2020) 8, no. 18, 4215–4222, 10.12998/wjcc.v8.i18.4215, 33024781. PMC7520787 33024781

[6] Maluf H. , King M. , DeLuca F. , Navarro J. , Talerman A. , and Young R. H. , Giant Multilocular Prostatic Cystadenoma, American Journal of Surgical Pathology. (1991) 15, no. 2, 131–135, 10.1097/00000478-199102000-00005. 1989460

[7] WHO Classification of Tumours Editorial Board , WHO Classification of Tumours: Urinary and Male Genital Tumours, 2022, 5th edition, International Agency for Research on Cancer.

[8] Do H. L. , Le N. H. D. , Chung T. T. , Le T. T. , and Truyen T. T. T. T. , Recurrent Giant Multilocular Prostatic Cystadenoma: A Case Report After a Decade in Vietnam, Urology Case Reports. (2026) 66, 103415, 10.1016/j.eucr.2026.103415, 41908852. PMC13019921 41908852

[9] Chughtai B. , Lee R. , Te S. , and Kaplan S. , Inflammation and Benign Prostatic Hyperplasia: Clinical Implications, Current Urology Reports. (2011) 12, no. 4, 274–277, 10.1007/s11934-011-0191-3. 21519898

[10] Werneburg G. , Shoskes D. , and de S. , Rapid Prostatic Regrowth and a Free-Floating Necrotic Bladder Mass Serving as a Nidus for Infection Following ThuLEP: Management With Morcellation and HoLEP, Urology. (2021) 148, e9–e10, 10.1016/j.urology.2020.11.041. 33279612

[11] Gupta S. , Martini A. , Wagaskar V. , and Tewari A. K. , Prostate Cancer in a Patient With Giant Prostatic Hyperplasia: The Robotic Approach to a Difficult Problem, Journal of Endourology Case Reports. (2019) 5, no. 3, 99–101, 10.1089/cren.2019.0024, 32775637. PMC7383426 32775637

[12] Datta M. , Hosenpud J. , Osipov V. , and Young R. H. , Giant Multilocular Cystadenoma of the Prostate Responsive to GnRH Antagonists, Urology. (2003) 61, no. 1, 225–1122, 10.1016/s0090-4295(02)02109-x, 12559312. 12559312

[13] Ohyama T. , Endo F. , Shimbo M. , and Hattori K. , Japanese Giant Benign Prostatic Hyperplasia: Sibling Cases, IJU Case Reports. (2022) 5, no. 4, 312–314, 10.1002/iju5.12467, 35795106. PMC9249644 35795106

[14] Shahed B. , Kosseifi F. , Dallongeville A. , and Durand X. , Holmium Laser Enucleation of A 696 cc Prostate (HoLEP): The Largest Reported in the Literature, Urology Case Reports. (2022) 45, 102203, 10.1016/j.eucr.2022.102203, 36092024. PMC9450164 36092024

[15] Scarcia M. , Moretto S. , Zazzara M. , Alba S. , Fiorentino A. , Ciampi L. , Ludovico E. , Calbi R. , and Ludovico G. M. , Robot-Assisted Extraperitoneal Radical Prostatectomy for Giant Multilocular Prostatic Cystadenoma: A Case Report and Literature Review, Urologia Internationalis. (2023) 107, no. 10-12, 983–987, 10.1159/000534176, 37913758. 37913758

